# *Learning the hard way: What COVID-19 teaches us about the social determinants of mental health among the urban poor*

**DOI:** 10.1186/s12889-025-25815-1

**Published:** 2026-01-09

**Authors:** Ignacio Diaz Concha, Roxana Valdebenito Montenegro, Francisca González López, Laura Orlando Romero, Alejandra Vives Vergara

**Affiliations:** 1 School of Public Health, Faculty of Medicine, Pontificia Universidad Católica de Chile, Santiago, Chile; 2 Departamento de Matemática, Universidad Técnica Federico Santa María, Valparaíso, Chile; 3 Centre for Sustainable Urban Development, CEDEUS, Santiago, Chile

**Keywords:** COVID-19 pandemic, Symtoms of depression/Mental health, Lockdown, Financial stress, Homeschooling, Urban-poor homemakers

## Abstract

**Background:**

The COVID-19 health crisis rapidly escalated into a broader social and economic emergency, affecting physical and mental health beyond the disease itself and placing a disproportionate burden on vulnerable populations. This study investigates the determinants of poor mental health related to COVID-19 among urban-poor homemakers residing in a social housing villa located on the outskirts of Santiago de Chile, a metropolitan city characterized by pronounced sociospatial segregation.

**Methods:**

Capitalizing on the ongoing RUCAS cohort study, we introduced a COVID-19 module into the biannual RUCAS survey to assess the associations between COVID-19-related social determinants of poor mental health and symptoms of depression during and after the 2020 lockdown among 413 homemakers. We describe the prevalence and distribution of COVID-19-related social determinants of poor mental health, and longitudinally examine their associations with depressive symptoms, measured using the Patient Health Questionnaire-2 (PHQ-2). Modified Poisson regression models were used to estimate prevalence ratios (PRs) and their corresponding 95% confidence intervals, including an interaction between the independent variable and the measurement wave for the longitudinal assessment.

**Results:**

During lockdown, symptoms of depression among homemakers were most strongly associated with students in the household facing difficulties in completing schoolwork during homeschooling (PR: 2.62; 1.60–4.30). After lockdown, the strongest associations were observed with employment (being out of the labor force (PR: 1.59; 1.04–2.44); receiving reduced income from work (PR: 1.95; 1.22–3.11)), and the financial situation of the household: indebtedness (wave 4: acquisition of new debts, PR: 1.48; 1.04–2.11; wave 5: low debt-payment capacity, PR: 1.44; 1.01–2.07), and food insecurity (wave 4, PR: 1.92; 1.38–2.65; wave 5, PR: 1.59; 1.12–2.26). Household conflicts over space were associated with depressive symptoms in both waves (wave 4, PR: 1.43; 1.01–2.02; wave 5, PR: 1.74; 1.23–2.46). No association was found with being or living with a COVID-19 case.

**Conclusions:**

In addition to the central role of financial stressors in shaping mental health outcomes during the COVID-19 pandemic, our results underscore the importance of home-schooling challenges as a critical source of psychological distress among peripheralized homemakers. These findings offer valuable insights for enhancing multisectoral preparedness in future crises, emphasizing the need to integrate social, economic, and educational support strategies for peripheralized urban-poor populations and to prevent or mitigate the deepening of existing social inequalities.

**Supplementary Information:**

The online version contains supplementary material available at 10.1186/s12889-025-25815-1.

## Background

The COVID-19 pandemic, initially a public health crisis, rapidly evolved into a social and economic crisis. It disproportionately affected already disadvantaged populations, where the most impoverished and overcrowded families and neighbourhoods experienced substantially higher rates of COVID-19 infection and death [1–3]. Confinement measures implemented to control viral transmission disrupted nearly all aspects of social and daily life, imposing a multifaceted burden on individuals and households and exacerbating many determinants of poor physical and mental health [4]. Although these effects varied both between and within countries, dramatic increases in unemployment and income loss, children missing school, and many families adopting confinement in the context of crowding and substandard housing implied socioeconomically deprived families were among the most severely affected [4].

Mental health impacts were identified early in the pandemic as a research priority, particularly among vulnerable populations [5]. A meta-regression of 48 studies with prepandemic baselines estimated a 27.5% increase in major depressive disorders and a 25.6% increase in anxiety disorders during the early phases of the pandemic [6]. Similarly, a WHO umbrella review of 21 meta-analyses published by October 2021 confirmed a significant global rise in mental health problems and emphasized the need for further research in low- and middle-income countries [7].

Social determinants of health played a central role in shaping these outcomes. Poor housing conditions, household composition, and caregiving responsibilities (e.g., living alone, with school-aged or preschool children, or managing homeschooling), as well as job and income loss, health-related fears, bereavement, and prolonged uncertainty, have all been linked to increased levels of depression and anxiety during the pandemic [7–14].

Among the population subgroups most affected by the social and psychological impacts of the pandemic are women, young adults, individuals with lower educational attainment and income, residents of deprived neighborhoods and areas hardest hit by the pandemic, ethnic minorities, and those with preexisting mental or physical health conditions [6, 15–17].

Like other metropolitan cities in South America, Santiago—the capital of Chile—is characterized by pronounced socioeconomic segregation, which is mirrored by overall health [18] and COVID-19 outcomes [19]. High population density, deteriorated neighborhoods, and poor-quality housing are especially critical in underserved urban peripheries, where precarious living conditions are compounded by low waged, precarious or informal jobs [20].

COVID-19 cases rose steadily in the country starting March 2020, peaking on June 14th, which is considered the first and deadliest pandemic wave in the country [21]. Lockdown measures and night curfews implemented to restrict mobility [22] resulted in a sharp downturn in economic activity [23], with a notable decline in informal jobs, especially those conducted in the open such as in open markets and public roads, resulting in significant financial strain for low-income families [22]. Communities responded to growing food insecurity by implementing community kitchens to provide daily meals to the most affected families [23]. School closures lasted for 259 school days, 147 during 2020, the longest among OECD countries [24], and relied heavily on remote schooling, which globally affected impoverished sectors the most [4].

Globally, online surveys have been widely used to assess the consequences of these measures. Low-income populations, however, are rarely reached given their limited internet access and digital literacy [9]. In this context, longitudinal studies previously in place, which could contact participants by telephone, represented a valuable opportunity to study more vulnerable populations [25, 26]. The “Regeneración Urbana, Calidad de Vida y Salud” (RUCAS) project is a longitudinal, multimethod study designed to evaluate the health impacts of an urban regeneration program in social housing villas in Chile [27]. Building on the RUCAS cohort and guided by the social determinants of health framework [28], we added a special module into the biannual RUCAS survey to gather data at both the individual and household levels. This module focused on the social and health consequences of the COVID-19 pandemic among urban-poor homemakers in a segregated, low-income community in the urban periphery of Santiago de Chile [27].

In this study, we describe these consequences during and after the first COVID-19 wave and lockdown of 2020 and explore the associations between these consequences and the mental health of homemakers, predominantly women, during and after lockdown. This unique context allows us to address a population underrepresented in COVID-19 research in general [28] and in Chile in particular, namely, peripheralized, urban-poor, homemakers in a social housing villa in a South American country. This can provide valuable lessons into the determinants of poor mental health in extreme situations, which are more likely today given the multiple consequences of climate change and global warming.

## Methods

### Design

Two-wave longitudinal study of urban-poor homemakers residing in a social housing villa in Puente Alto, in the periphery of Santiago de Chile. It is embedded in waves 4 (w4) and 5 (w5) of the RUCAS project [27], a longitudinal study devised to study the health and health-related effects of a governmental program of urban regeneration in social housing complexes. The baseline RUCAS sampling frame included all households in the villa that were included in the government roster for renovation, excluding those representing security concerns, dwellings without inhabitants or those used for non-residential purposes [27]. The resulting baseline sample included 718 households (response rate of 89%).

**Fig. 1:**
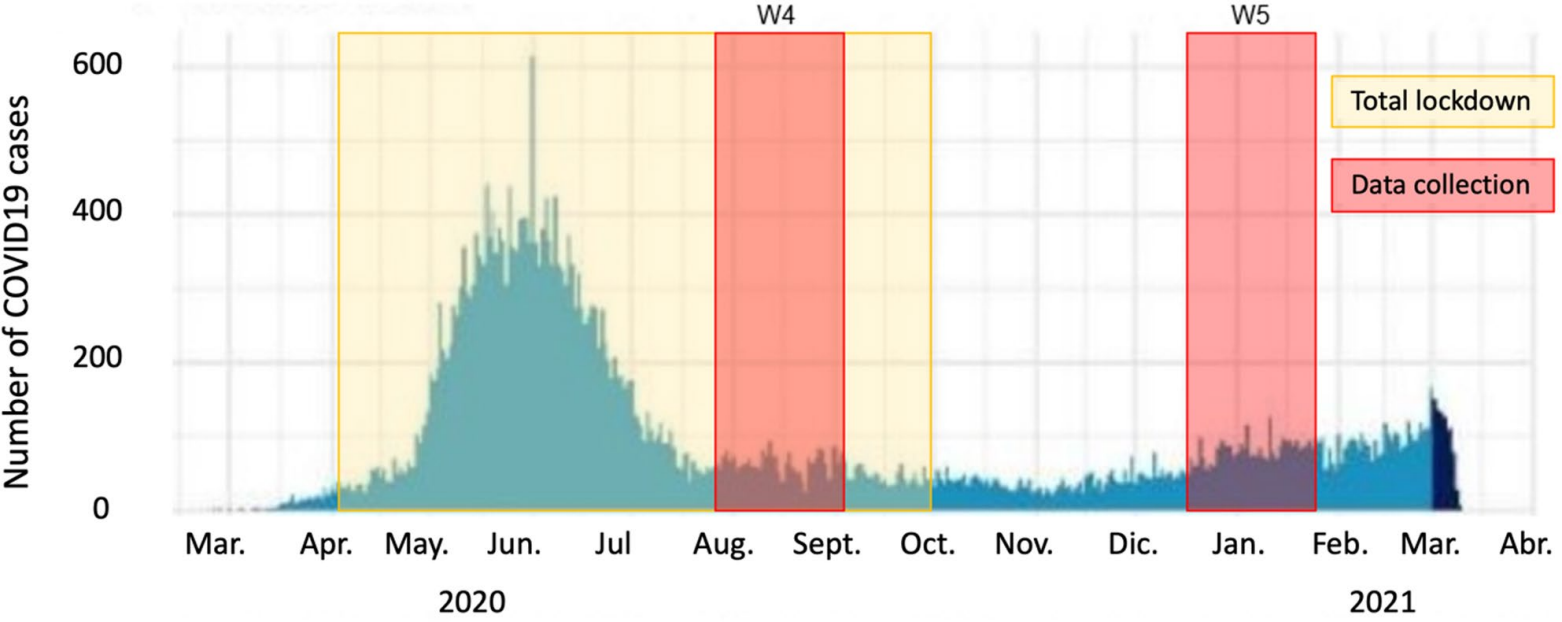
Timeline. Number of COVID-19 cases by date of symptom initiation. March 2020–April 2021, Puente Alto, Santiago de Chile. Source: Adapted from "*Informe Epidemiológico MINSAL Chile*" (2021). In yellow, total lockdown period (encompassing strict stay-at-home orders, curfew, isolation of cases, school closures, and cordons sanitaires); in red, period of data collection (w4: August 11 to September 17, 2020; w5: January 07 to February 12, 2021)

Data collection took place during total lockdown (w4: August 11–September 17, 2020) and five months after lockdown had been lifted (w5: January 07–February 12, 2021). Figure 1 shows when data were collected in relation to the evolution of daily COVID-19 cases and lockdown in Puente Alto, the municipality where the study community is located. W4 took place after the first, steepest wave of COVID-19 cases and during lockdown. W5 took place after a period of relatively low incidence of COVID-19 infection and the release of lockdown.

### Sample

The study sample included homemakers aged 18 years or older who participated in both study waves, w4 and w5 (*n* = 413). For this study, participants were interviewed by phone via contact numbers collected in previous survey waves.

The RUCAS project (ID 170727004) and the RUCAS COVID project (ID 200521011) were approved and annually revised by the Institutional Review Board of the Faculty of Medicine of Pontificia Universidad Católica de Chile. Participation was voluntary and confidential, and informed consent was signed at enrolment. In addition, all the subjects provided informed consent for their participation in the RUCAS-COVID-19 telephone survey waves. The data have been deidentified, and their transfer and storage adhere to the usual standards of privacy and data protection.

### Variables

#### Mental health outcomes

1. Symptoms of Depression: Measured with the 2-item version of the Patient Health Questionnaire–2 (PHQ-2), a self-report questionnaire used to screen for major depressive disorders by exploring the frequency of the core symptoms of depression, depressed mood and anhedonia during the preceding two weeks [30]. The PHQ-2 demonstrated adequate sensitivity (0.72), specificity (0.85) [31] and stability [32]. It was validated in Chile in low-income, urban, primary health-care patients, against the PHQ-9 [33]. PHQ-2 score ranges from 0 to 6; we used a cut-off score of 3 or higher, which has been established as the optimal cut-off to screen for depression in several countries and in low-income populations in Chile [34].
2. Mood decline: In w4, respondents assessed their perceived current mood relative to that before the pandemic; in w5, they were asked to assess their mood relative to three months prior, that is, after lockdown was lifted. Responses on a 5-pointLikert scale ranged from much worse to much better and were dichotomized into (i) worse (worse and much worse) and (ii) same or better (same, better or much better).

#### COVID-19-related determinants of mental health

Following the framework of the social determinants of health adapted to the COVID-19 context [28], our independent variables are determinants of mental health especially relevant for women homemakers in low-income households. When indicated, the reference periods are as follows: w4: March–September 2020; w5: October–January 2021.

#### Caregiving: household load of domestic and unpaid care work

Household composition: The burden of care was assessed by the presence of dependents in the household: toddlers (≤ 2 years), children (< 15 years), and older adults (≥ 65 years). For the latter analysis, we excluded 56 respondents who were themselves aged 64 years or more.

 Students´ school-work completion: after transfer to remote schooling on March 16^th,^ 2020 [34]. In w4, we assessed the general situation of all schoolchildren in the household since lockdown, using a single question: *“Considering the school-aged children or youth*,* would you say that they have been able to complete their homework/activities during the COVID-19 crisis?”*, dichotomized into achieved (same or better than usual) and not achieved (less than usual or not at all). In w5, we assessed achievement for each school child individually: “*Considering children or youth of school age*,* was [NAME] able to complete the homework and activities of the school year?”* which we recoded and dichotomized as achieved (all students in the household completed their schoolwork) and not achieved (at least one student in the household did not -or only partially- complete schoolwork). Response categories were modified in w5, as it was not meaningful to compare with the prepandemic ‘usual’ after 10 months of school closures. We included only households with school students in this analysis (n = 207).

#### Housing and relationships between household members

Living alone (yes/no): identifies one-person households, i.e., individuals living by themselves.

 Overcrowding (>2 residents per bedroom): associated with inadequate space within the dwelling for living, sleeping and household activities [35] and potentially exacerbated by confinement in these 42 m² dwellings. Given the small size of bedrooms in these dwellings [36], we used a lower threshold than the social policy standard of 2.5 residents per bedroom.

Household relationships are related to the scarcity of indoor space, assessed with 5-point Likert scales (never to always) as (1) being able to hold a private conversation and (2) having conflicts over the use of (indoor) space, grouped as always/almost always, sometimes, and never/almost never. For the latter (conflicts over the use of space), only respondents who did not live alone in either w4 or w5 were included (*n* = 361), given it is unlikely that such conflicts occur if living alone.

#### Employment conditions during the COVID-19 pandemic

Employment trajectory during the reference period: a 4-category variable indicating whether the respondent had been (1) always employed, (2) recently employed (currently working), (3) lost their job (currently not working), or (4) never employed.

Employment status at the time of the survey: a 4-category variable indicating whether the respondent was (1) employed without income loss, (2) employed but lost income, (3) unemployed, or (4) out of the labour force.

#### Household financial situation (economic resources)

Indebtedness during the reference period (w4): assessed on the basis of whether households acquired new debts (debt, loan, or credit) as a result of the COVID-19 crisis. Responses were categorized as follows: yes (new debts acquired), no new debts (but had prior debts), and no debts (neither new nor prior).

Problematic indebtedness (w5) was assessed on the basis of the reported likelihood of households being able to repay their debts. Responses were categorized as follows: unlikely (somewhat likely, unlikely, or not at all likely), likely (likely or very likely), or no debts.

Financial distress: Perceived degree of financial distress in the 12 months preceding w5 (little/none; moderate; high/severe).

Food insecurity: A single item adapted from the Food Insecurity Experience Scale (FIES) [37], which measures moderate food insecurity (whether anyone in the household skipped a meal because there was not enough money or other resources to obtain food) during the COVID-19 crisis, dichotomized into yes (always, most of the time, or sometimes) or no (never).

#### COVID-19 infection during the reference period

Respondent: being a confirmed case with a positive polymerase chain reaction (PCR) test, a medical diagnosis, or hospitalization for COVID-19. In w5, medical diagnosis was not assessed.

Dwelling: At least one resident in a dwelling was diagnosed with COVID-19, using the same criteria indicated above.

#### Covariates

We adjust our models by three main covariates, which are potential confounders in the association between mental health and the COVID-19-related social determinants of health: gender (women, men), age (continuous and categorical) and socioeconomic position, measured as the number of completed years of formal education (< 4, 4–7, 8–12, > 12).

### Analysis

We perform a descriptive analysis of the determinants of mental health and mental health variables during each wave. Then, by means of modified Poisson regressions, we calculate prevalence ratios (PRs, 95% CIs) (PR) [38]. We use prevalence ratios instead of odds ratios because prevalence rates allow a more straightforward interpretation of results when the prevalence of the outcome is high (above 10%) [39]. We used robust variance estimation to account for the overestimation of the standard error [39].

We constructed a model for each independent variable, adjusted by covariates: gender, age (continuous), and educational level of the homemaker.

$$\begin{aligned}\:\mathrm{log}\left(\pi\:\right)&=\:{\beta\:}_{0}+\:{\beta\:}_{1}\bullet\:I\left({X}_{i}\right)+\:{\beta\:}_{2}\bullet\:I\left(Sex\right)\\&+\:{\beta\:}_{3}\bullet\:Age+\:{\beta\:}_{4}\bullet\:I\left(Education\right)\end{aligned}$$

To incorporate the longitudinal component of the study, we included an interaction term between the independent variable and the measurement wave in the previous model:

$$\begin{aligned}\:\mathrm{log}\left(\pi\:\right)=\:{\beta\:}_{0}&+\:{\beta\:}_{1}\bullet\:I\left({X}_{i}\right)+\:{\beta\:}_{2}\bullet\:I\left({X}_{i}\right)\bullet\:I\left(Wave\right)\\&+\:{\beta\:}_{3}\bullet\:I\left(Sex\right)+\:{\beta\:}_{4}\bullet\:Age\\&+\:{\beta\:}_{5}\bullet\:I\left(Education\right)\end{aligned}$$

where π represents the probability of poor mental health; X_i_ represents each of the independent variables; and the function I indicates that the variable is categorical.

All analyses were performed via Stata 14 [40].

## Results

Table 1 presents sociodemographic and household characteristics of the study sample in waves 4 and 5. The majority of participants were women (85%), predominantly aged 45–64 years (61%), with most having completed 8–12 years of formal education (67%). Regarding unpaid care work, 12% lived with a toddler (8% in wave 5), 45% with a child under 15, and 14% with an older adult (65+). In those households with school-aged children or adolescents (*n* = 205), 55% reported that students experienced difficulties in completing schoolwork during wave 4, a figure that declined to 25% in wave 5. Reports of conflicts over the use of space and difficulties in holding a private conversation increased in wave 5, when lockdown measures had been relaxed.

**Table 1:** Sample description and distribution of study variables (w4 and w5). August 2020 and January 2021

		w4		w5	
Date of survey application		11/08/2020-17/09/2020		07/01/2021-12/02/2021	
		n	%	n	%
Homemaker sociodemographic characteristics					
	Men	61	14.8	-	-
	Women	352	85.2	-	-
Age groups (years)	18-24	14	3.4	5	1.2
	25-44	101	24.5	105	25.4
	45-64	251	60.8	247	59.8
	≥ 65	47	11.4	56	13.6
Age (years)	Mean (SD)	49.9 (12.3)		50.9 (12.2)	
Completed years of formal education	< 4	32	7.9	-	-
	4 to 7	84	20.7	-	-
	8 to 12	270	66.7	-	-
	>12	19	4.7	-	-
Mental health outcomes					
Symptoms of depression	No	291	70.6	295	71.4
	Yes	121	29.4	118	28.6
Mood decline	Same or better	166	40.5	303	73.5
	Worse	244	59.5	109	26.5
Household load of domestic and unpaid care work					
Toddlers (≤2 years)	No	365	88.4	380	92.0
	Yes	48	11.6	33	8.0
Children (<15 years)	No	226	54.7	232	56.2
	Yes	187	45.3	181	43.8
Older adults (≥65 years)	No	343	85.8	330	83.1
	Yes	57	14.3	67	16.9
Students´ schoolwork completion*	Achieved	93	45.4	135	75.0
	Not achieved	112	54.6	45	25.0
Relationships between household members					
Living alone	No	365	88.4	365	88.4
	Yes	48	11.6	48	11.6
Overcrowding	No	351	85.4	357	86.4
	Yes	60	14.6	56	13.6
Being able to hold a private conversation	Always/almost always	161	39.5	135	33.0
	Sometimes	58	14.2	33	8.1
	Never/almost never	189	46.3	241	58.9
Conflicts over the use of (indoor)	Never/almost never	234	65.0	230	63.2
space**	Sometimes	45	12.5	58	15.9
	Always/almost always	81	22.5	76	20.9
Employment during COVID-19					
Employment trajectory	Always occupied	143	36.6	146	36.9
	Found a job	9	2.3	58	14.6
	Loss a job	86	22.0	46	11.6
	Never occupied	153	39.1	146	36.9
Employment status	Occupied who didn't lose income	64	16.4	125	30.8
	Occupied who lost income	77	19.7	80	19.7
	Unemployed	20	5.1	20	4.9
	Out of the labour force	230	58.8	181	44.6
Household financial situation					
Indebtedness***	No debts	206	50.4	-	-
	No new debts	117	28.6	-	-
	Acquired new debts	86	21.0	-	-
Problematic indebtedness****	No debts	-	-	286	69.8
	Likely	-	-	54	13.2
	Unlikely	-	-	70	17.1
Financial distress****	Little/none	-	-	105	25.4
	Moderate	-	-	223	54.0
	High/severe	-	-	85	20.6
Food insecurity	No	356	87.0	355	86.0
	Yes	53	13.0	58	14.0
COVID-19 infection					
Homemaker	No case	384	93.4	402	97.3
	Case	27	6.6	11	2.7
Household member	No case	366	88.8	390	94.4
	Case	46	11.2	23	5.6

* Only households with school students are considered (n=207 – missing data)

** Only households with more than one member (n=365 – missing data)

*** Variable available only in w4

**** Variables available only in w5

In wave 4, 19.7% of those in a paid job had lost income compared to before the pandemic; in wave 5, 50.7% were employed (19.7% had lost income). The proportion out of the labour force was reduced by 14% points between w4 (59%) and w5 (45%). In w4, 21% of households had acquired new debt due to the COVID-19 crisis. In w5, 17% had debts that they were unlikely to be able to pay. Financial stress in the past 12 months (measured in w5) was high or very high for 21%. Moderate household food insecurity was reported by 13% in w4 and 14% in w5. Approximately 7% of the participants had been diagnosed with COVID-19 by w4, and an additional 2.7% were diagnosed by w5. At the household level, at least one case of COVID-19 was reported in 11.2% of households by w4 and an additional 5.6% by w5.

Table 2 shows that in wave 4, the prevalence of depressive symptoms among women was approximately twice that among men. By wave 5, it slightly decreased in women but increased by 5% points in men, significantly narrowing the gender gap. Similarly, mood decline decreased by more than half in women between waves 4 and 5 (36% points), whereas the reduction in men was of only 36% (14% points). In terms of age, symptoms of depression followed an inverse gradient in both waves, with higher rates among younger respondents. In wave 5, symptoms of depression increased by 4% points among the youngest group (18–24 years) and among the oldest group (65+), who reported the largest decline in mood during this wave. In wave 4, symptoms of depression were more prevalent among individuals with middle and middle–high levels of education, whereas mood decline increased progressively from low to high educational levels. By wave 5, this pattern reversed: the prevalence of symptoms of depression was highest among those with the lowest and highest education levels, and mood decline followed a gradient from high to low education.

**Table 2:** Prevalence of symptoms of depression and mood decline according to homemaker sociodemographic in w4 and w5

			w4						w5					
		Symptoms of depression			Mood decline			Symptoms of depression				Mood decline		
		Prevalence	PR	95% CI	Prevalence	PR	95% CI	Prevalence		PR	95% CI	Prevalence	PR	95% CI
Gender	Men	16.4%			39.3%			21.3%				25.0%		
	Women	31.6%	1.93	1.08–3.45	63.0%	1.60	1.16–2.21	29.8%		1.59	0.91–2.78	26.7%	1.16	0.71–1.89
Age group	18–24	35.7%			50.0%			40.0%				20.0%		
(years)	25–44	32.0%	0.81	0.37–1.75	60.0%	1.12	0.64–1.95	28.6%		0.70	0.21–2.33	19.2%	0.92	0.15–5.78
	45–64	30.7%	0.76	0.36–1.60	61.4%	1.19	0.69–2.04	29.4%		0.77	0.24–2.52	26.8%	1.33	0.21–8.29
	≥ 65	22.9%	0.66	0.27–1.65	56.4%	1.20	0.66–2.16	26.6%		0.57	0.15–2.09	33.0%	1.53	0.23–10.03
Completed years of formal education	< 4	21.9%			56.3%			31.3%				31.3%		
	4 to 7	27.4%	1.14	0.54–2.38	57.8%	0.96	0.67–1.37	22.6%		0.65	0.34–1.24	31.0%	1.00	0.53–1.87
	8 to 12	32.0%	1.33	0.67–2.62	61.2%	1.06	0.77–1.47	29.9%		0.85	0.49–1.48	24.8%	0.88	0.48–1.60
	> 12	21.1%	0.81	0.27–2.41	63.2%	1.11	0.68–1.80	31.6%		0.87	0.36–2.07	21.1%	0.85	0.29–2.53

Table 3. shows the adjusted prevalences in longitudinal models that include an interaction between time and the independent variable. Living with toddlers or children under 15 years of age was not significantly associated with symptoms of depression during lockdown, especially after it was lifted. In contrast, living with older adults was associated with depressive symptoms in wave 5 (PR: 1.41; 0.94–2.10). Having a student in the household experimenting difficulties completing their homework was strongly associated with depressive symptoms during wave 4 (PR: 2.62; 1.60–4.30) and moderately associated with depressive symptoms during wave 5 (PR: 1.56; 0.98–2.46).

**Table 3.** Prevalence of symptoms of depression and mood decline across COVID-19-related determinants of mental health and prevalence ratios (95% CI), adjusted by sex, age and educational level (w4 and w5)

		**W4**						W5					
		Symptoms of depression			Mood decline				Symptoms of depression			Mood decline	
		Prevalence	PR	95% CI	Prevalence	PR	95% CI	Prevalence	PR	95% CI	Prevalence	PR	95% CI
Household load of domestic and unpaid care work													
Toddlers (≤ 2 years)	No	28.0%			59.4%			28.7%			27.1%		
	Yes	39.6%	1.32	0.9 - 1.95	60.4%	1.01	0.79 - 1.29	27.3%	0.92	0.51 - 1.67	18.8%	0.70	0.33 - 1.47
Childen (< 15 years)	No	27.0%			62.9%			28.0%			26.5%		
	Yes	32.3%	1.11	0.79 - 1.58	56.7%	1.11	0.93 - 1.33	29.3%	0.99	0.7 - 1.42	22.8%	0.79	0.56 - 1.12
Older adults (>64 years) *	No	29.2%			58.7%			27.3%			22.8%		
	Yes	30.0%	1.23	0.8 - 1.9	63.8%	1.11	0.9 - 1.38	33.7%	1.41	0.94 - 2.1	32.5%	1.30	0.89 - 1.89
Students´ school-work completion**	Achieved	17.2%			49.5%			26.7%			25.2%		
	Not achieved	42.9%	2.62	1.6 - 4.3	72.3%	1.47	1.16 - 1.86	40.0%	1.56	0.98 - 2.46	24.4%	0.96	0.53 - 1.74
Relationships between household members													
Live alone	No	30.8%			60.6%			29.3%			25.8%		
	Yes	18.8%	0.65	0.34 - 1.27	51.1%	0.87	0.63 - 1.19	22.9%	0.88	0.49 - 1.58	31.3%	1.19	0.73 - 1.94
Overcrowding	No	28.2%			57.9%			27.2%			26.1%		
	Yes	35.0%	1.19	0.81 - 1.75	68.3%	1.15	0.95 - 1.4	37.5%	1.41	0.96 - 2.06	29.1%	1.14	0.73 - 1.78
Being able to hold a private conversation	Always/almost always	26.1%			55.0%			19.3%			20.7%		
	Sometimes	27.6%	1.03	0.63 - 1.68	70.7%	1.25	1 - 1.56	18.2%	0.93	0.42 - 2.06	27.3%	1.30	0.69 - 2.47
	Never/almost never	32.8%	1.26	0.9 - 1.76	60.8%	1.13	0.95 - 1.36	35.3%	1.73	1.17 - 2.55	28.8%	1.35	0.91 - 1.98
Conflicts over the use of (indoor) space***	Never/almost never	26.1%			57.7%			24.3%			27.9%		
	Sometimes	35.6%	1.32	0.84 - 2.06	53.3%	0.91	0.68 - 1.2	29.3%	1.25	0.79 - 1.98	20.7%	0.76	0.44 - 1.32
	Always/almost always	39.5%	1.43	1.01 - 2.02	72.8%	1.24	1.04 - 1.48	43.4%	1.74	1.23 - 2.46	23.7%	0.84	0.54 - 1.33
Employment during COVID-19													
Employment trajectory	Always occupied	28.0%			56.6%			26.7%			21.9%		
	Found a job	22.2%	0.99	0.27 - 3.58	33.3%	0.67	0.27 - 1.66	22.4%	0.90	0.52 - 1.56	19.3%	0.92	0.5 - 1.7
	Loss a job	39.5%	1.39	0.96 - 2.01	69.4%	1.21	0.99 - 1.48	34.8%	1.34	0.84 - 2.13	37.0%	1.67	1.03 - 2.71
	Never occupied	28.1%	1.01	0.7 - 1.45	59.9%	1.04	0.86 - 1.26	29.5%	1.05	0.73 - 1.53	30.8%	1.33	0.89 - 1.98
Employment status	Occupied who didn't lose income	1.0%			51.6%			18.4%			21.6%		
	Occupied who lost income	33.8%	1.65	0.95 - 2.84	61.0%	1.21	0.90 - 1.63	36.3%	1.95	1.22 - 3.11	20.3%	0.94	0.54 - 1.64
	Unemployed	40.0%	1.91	0.96 - 3.81	55.0%	1.11	0.71 - 1.72	35.0%	1.88	0.93 - 3.79	25.0%	1.17	0.52 - 2.64
	Out of the labour force	30.4%	1.42	0.86 - 2.34	62.7%	1.22	0.94 - 1.57	30.9%	1.59	1.04 - 2.44	32.6%	1.44	0.97 - 2.16
Household financial situation													
Indebtedness****	No debts	26.2%			55.1%								
	No new debts	27.4%	1.06	0.72 - 1.54	65.0%	1.17	0.97 - 1.4						
	Acquired new debts	39.5%	1.48	1.04 - 2.11	64.0%	1.16	0.95 - 1.42						
Problematic indebtedness*****	No debts							25.9%			25.9%		
	High payment capacity							27.8%	0.99	0.59 - 1.64	34.0%	1.21	0.78 - 1.87
	Low payment capacity							38.6%	1.44	1.01 - 2.07	24.3%	0.99	0.62 - 1.56
Financial distress*****	Little/none							21.0%			23.1%		
	Moderate							24.2%	1.35	0.85 - 2.16	24.2%	1.63	1.01 - 2.64
	High/severe							49.4%	1.06	0.73 - 1.56	36.5%	1.31	0.87 - 1.97
Food insecurity	No	26.4%			58.3%			26.5%			25.6%		
	Yes	49.1%	1.92	1.38 - 2.65	69.8%	1.22	1 - 1.49	41.4%	1.59	1.12 - 2.26	31.6%	1.23	0.8 - 1.88
COVID-19 infection													
Homemaker	No case	29.7%			59.8%			29.1%			26.4%		
	Case	25.9%	0.84	0.43 - 1.62	53.8%	0.90	0.64 - 1.27	9.1%	0.31	0.05 - 1.97	27.3%	1.00	0.37 - 2.73
Household member	No case	29.5%			59.5%			29.2%			26.2%		
	Case	28.3%	0.92	0.56 - 1.5	60.0%	0.99	0.78 - 1.27	17.4%	0.61	0.25 - 1.47	30.4%	1.19	0.62 - 2.3

* Only homemakers aged less than 65 years and not living alone are considered (n= 357)

** Only households with school students are considered (n=207- missing data)

*** Only households with more than one member (n=365 – missing data)

**** Variable available only in w4

***** Variables available only in w5

Living alone and overcrowding were not significantly associated with depressive symptoms in w4, but overcrowding was associated with depressive symptoms in w5 (PR: 1.41; 0.96–2.06). Being unable to hold private conversations at home was only associated with depressive symptoms after lockdown had been relaxed (PR: 1.73; 1.17–2.55). Conflicts over space within the dwelling was associated with depressive symptoms during lockdown (PR always: 1.43; 1.01–2.02) and more so after lockdown (PR: 1.74; 1.23–2.46).

In w4 symptoms of depression were associated with having lost a job (PR: 1.38; 0.95–2.01), and with being unemployed (PR = 1.91; 0.96–3.81) or employed but having lost income (PR = 1.65; 0.95–2.84). In w5, only current employment status was associated with depressive symptoms: employed but having lost income (PR: 1.95; 1.22–3.11), unemployed (PR: 1.88; 0.93–3.79), or out of the labour force (PR: 1.59; 1.04–2.44). Mood decline was associated only with having lost a job or being out of the labour force, especially in w5 (PR: 1.67; 1.03–2.7).

The household’s financial situation was associated with symptoms of depression in both waves: acquiring new debts in w4 (PR: 1.48; 1.04–2.11) and a low capacity to repay debts in w5 (PR: 1.44; 1.01–2.07); household food insecurity in w4 (PR: 1.92; 1.38–2.65) and w5 (PR: 1.59; 1.12–2.26); and moderate financial distress, measured only in w5 (PR: 1.63; 1.01–2.64).

## Discussion

This study describes the social and economic consequences of the COVID-19 pandemic and their associations with symptoms of depression among urban-poor homemakers in the urban periphery of Santiago, Chile, during and after total lockdown. The study yields the following main findings: (1) the prevalence of symptoms of depression among homemakers changed as the pandemic evolved; (2) the determinants most strongly associated with depressive symptoms during lockdown were the difficulties of school-age youth in completing schoolwork; and (3) the determinants most strongly associated with depressive symptoms after lockdown were financial, such as a lack of employment, having diminished income from work, problematic indebtedness, food insecurity, and financial stress. Neither personal diagnosis of COVID-19 nor having a household member diagnosed was associated with depressive symptoms.

### General dynamics

Psychological distress and anxiety are normal initial responses to unfamiliar and threatening new situations, typically followed by recovery [41, 42]. Several early COVID-19 studies described a pattern of increase and subsequent decrease in symptoms of depression and anxiety [41, 43], although some later meta-analyses reported that these effects were small [44]. Following this pattern, in Chile, Duarte et al. (2022) [45] reported high levels of psychological distress during the first wave of COVID-19 (22.6%) and higher levels immediately afterwards, during total lockdown (27%). In our study, we observed a 29.4% prevalence of depressive symptoms during total lockdown and (28.6%) after lockdown was lifted, which is in line with the general pattern just described.

However, collective trauma impacts groups differently [46]: we find that women and young adults had the highest prevalence of depressive symptoms during lockdown, whereas older adults had the lowest prevalence, a finding that is largely consistent with previous studies in Chile [45, 47], other Latin American countries [47], and abroad [9, 44, 48]. Generally, lockdown impacted women’s daily lives with an increase in the complexity of, and time dedicated to, domestic and care work. Among young adults, loneliness, low income, unemployment, or not being in school may explain their poor mental health [6, 15, 16], whereas stay-at-home orders may have been less disruptive to older adults. Finally, the higher prevalence of depressive symptoms among middle-educated individuals during lockdown was also described by Salas et al. [47] in Chile and other Latin American countries, although the reasons remain unclear.

Additionally, not all studies describe a subsequent decline or return to prepandemic levels [17, 48, 49] or a single pattern of change over time [50]. In England, Jackson et al. [51] reported that psychological distress increased steadily over the two years of the pandemic among younger adults but not among those aged 65 years or older. Instead, in the United States, Lin et al. [52] reported long-term implications for the mental health of older adults more than two years after the pandemic. Some studies with longer follow-up periods have reported fluctuations that mirror changes in confinement measures and pandemic intensity [12, 44, 53]. Studies in the UK and Catalonia reported the greatest improvements in mental health among women, but to a lesser extent, among younger people and people of lower socioeconomic position, once cases had decreased and lockdowns had been relaxed [17, 43]. In our study, the prevalence of symptoms of depression decreased after lockdown among women, middle-aged adults, and those with mid-level education but increased among men, younger adults and older adults, particularly among those with the lowest and highest levels of education. It is likely that the underlying determinants are different and that, for example, the impact of sustained job or income loss may partially explain the increase in depressive symptoms among men in w5, whereas the burden of house and care work during lockdown may partially explain the reduction, although mild, in the prevalence of depressive symptoms among women once lockdown was lifted. In fact, the variables associated with symptoms of depression are consistent with previous knowledge (e.g., unemployment, financial stress), highlighting the strong contextual underpinnings of such patterns.

### Burden of care: household domestic and unpaid care work

Increased household chores and domestic work among carers and homemakers during lockdown have been described as key COVID-19-related stressors [54], with women bearing the brunt of this burden. We measured this load indirectly as household composition, and observed that living with toddlers and older adults, but no youth, was associated with symptoms of depression during lockdown. The association between depressive symptoms and the presence of dependents in the household during COVID-19 has been previously described in Spain and among women in Latin America [9, 47], with an English study reporting higher initial levels of anxiety and depressive symptoms among those living with older adults than those living with minors [15]. By our second measurement, we observe no association between symptoms of depression and living with toddlers, in line with several studies showing that adults living with children exhibited early improvements in mental health following reductions in COVID-19 cases and relaxation of lockdowns [17]. In contrast, the association with living with older adults persisted, which may be related to older adults being at increased risk all throughout the pandemic.

While living with minors does not appear to be a critical stressor per se [52], our study found that living with youth experiencing difficulties completing schoolwork was the factor most strongly associated with depressive symptoms among homemakers during lockdown, despite a rather small sample size for the corresponding analysis (*n* = 207). This is a novel and important finding, for which evidence remains scarce. In Chile, school performance during lockdown has been linked to students’ emotional well-being; however, there is limited evidence regarding the impact of children’s academic difficulties on the mental health of adults in the household.

Related findings have been described elsewhere, showing, for example, that among personal, family and environmental factors, the main predictor of maternal psychological distress during lockdown was school closures [55] and that women who spent more time on childcare and home schooling reported greater feelings of overwhelm and decline in mental health [17], especially among those with marginalization experiences, low socioeconomic status or prior mental illness [578, 56].

During lockdown, caregivers juggled domestic and care work with home schooling [57], which requires adult support and environmental conditions that low-income families generally lack [58–60]. For example, in England, a lack of access to electronic devices and adequate workspace was significantly associated with poor mental wellbeing of parents or carers [61]. In addition, lockdown has resulted in psychological distress among children and adolescents [62]– [63], which, compounded by a stressful environment in the home and increased parenting stress, can increase negative parent‒child interactions and family functioning and further affect children’s well-being and students’ ability to complete schoolwork [64]– [65].

Furthermore, in Chile, remote schooling took a strong online approach [34, 66] which requires specific skills, but a 2018 study revealed that 90% of eighth-grade students lacked the skills to independently use a computer [59]; most schools were unprepared for distance teaching, and lower-income families had limited access to electronic devices and a reliable internet connection. The physical distribution of study guides and textbooks was an alternative solution, albeit hampered by mobility restrictions [67]. All the above imply reduced learning time, which can be especially distressing for low-income families, who value education as the main way out of poverty. In our study, when lockdown had been lifted and the school year had finished, the reported completion of schoolwork was much higher than that in w4 (Table 1), and the association with depressive symptoms was substantially reduced. This may reflect an actual improvement in, or reduced caregiver burden or awareness of, students’ school performance [68] and a reduction in uncertainties concerning schooling outcomes.

### Household relationships

Qualitative studies in Chile report that women in the context of strict mobility restrictions describe that overcrowding and living with schoolchildren or older adults increased their experience and perception of lack of space, which, in normal periods, are attenuated because household members spend time away from home [69]. During the COVID-19 pandemic, the demands placed on the home increased significantly, as it had to accommodate schooling, caregiving, and work [41]. These results align with previous findings that overcrowding and a perceived lack of space in the home were associated with psychological distress during the COVID-19 lockdown [52]. In our study, however, conflicts over indoor space were only slightly lower after lockdown was lifted, possibly because in social housing neighborhoods, the extreme lack of space pushed families to spend time outdoors. Being unable to hold a private conversation, a more frequent complaint, was more prevalent in w5 than in w4, suggesting that time spent together during lockdown may have given adults new opportunities for interaction. Together with overcrowding, both measures were more strongly associated with depressive symptoms after lockdown was lifted, possibly reflecting the persistence of the most severe cohabitation-related stressors and their mental health consequences beyond the lockdown period. In contrast to other studies that reported an association between perceived loneliness and psychological distress e.g., Duarte et al., 2022 [45], our findings revealed no significant link between living alone and symptoms of depression.

### Employment during COVID-19

Economic uncertainty and financial strain emerged as major stressors during the COVID-19 pandemic in Chile [70], with concerns about job loss, reduced income, and the ability to meet basic needs being strongly associated with psychological distress in adults [45]. We find that having recently lost a job, being unemployed, out of the labour force or in a lower earning job at the time of the survey was associated with depressive symptoms (and mood decline) in both waves, which is consistent with the well-known consequences of job and income loss [71] and with previous research describing such consequences both in the short and longer term after COVID-19 [17, 52, 66]. Some of these associations were stronger in w5, which may be related to unfulfilled expectations of employment and financial recovery once lockdown was lifted, especially among men. In Latin America, economic crises tend to result in persistent employment loss and a slow recovery of formal jobs, especially among low-skilled workers [72]. In Chile, the accelerated destruction of jobs during total lockdown especially affected informal jobs and was followed by a slow recovery of employment, which returned to prepandemic levels only by the end of 2021 [73]. In addition, unemployment and financial problems have been described as persistent determinants of psychological distress in adults, even after the stressors that cause them have receded [52, 74].

### Financial situation of the household

Economic shocks can increase the prevalence of common and more serious mental disorders, especially among those enduring serious economic hardship, as observed before and during the COVID-19 pandemic [68, 75–77]. In line with the above findings, in our study, financial hardship, expressed as food insecurity (PRw4: 1.92; 1.38–2.65; PRw5: 1.59, 1.12–2.26), indebtedness (PRw4: 1.48; 1.04–2.11) and problematic indebtedness (PRw5: 1.44; 1.01–2.07), were associated with homemaker depressive symptoms both during and after lockdown. Studies abroad have described associations between financial and food insecurity and mental health, particularly among parents [17]. Problematic indebtedness is likely to be a serious source of psychological distress [78], especially in times of high unemployment. In fact, a previous study suggests that it may be a key determinant of increased suicide mortality during economic crises in Chile [79].

Notably, the proportion recurring to loans in our sample is smaller than the national average estimated by social survey data (21% vs. 45%). This may be related to the limited borrowing capacity of low-income households. According to the Chilean COVID-19 social survey, as of July 2020, the most frequent economic strategies deployed by lower-income households during the pandemic were the reduction of spending on food and basic services and the recourse to household savings and loans from relatives or acquaintances [80]. In line with our findings, by December 2021, the lowest SES quintile had the lowest prevalence of indebtedness in Chile, despite having increased their indebtedness the most compared with that before the pandemic and having the most trouble paying their debts [80].

### COVID-19 infection

Contrary to expectations, we found no association between depressive symptoms or mood decline and COVID-19 infection, despite the fact that, by wave 4, vaccines were not yet available, and health services were under critical strain, especially those serving low-income populations. A multicountry study revealed that stress was more strongly related to confinement, family dynamics and financial hardship than to health-related concerns, suggesting that reduced exposure due to lockdown measures may have mitigated anxiety related to infection [22]. Moreover, individuals experiencing greater distress about COVID-19 may have adopted more cautious behaviours, thereby lowering their risk of infection and weakening the observed associations [81]. Similarly, a large transcontinental study reported a significant yet minimal association with COVID-19-related deaths [82] [83]. And, in contrast to findings from other countries in the region [47], an online study conducted in Chile revealed no association between self-rated health and either having had COVID-19 personally or living with someone who had contracted the virus [84]. Other studies have described that COVID-19 symptomatology, diagnosis, or, among women, cohabiting with someone with a COVID-19 diagnosis or symptomatology, as well as bereavement, were associated with anxiety or psychological distress over the following year [9, 13], associations which shorter-term studies may fail to find.

Additionally, because we are restricting our measure to confirmed COVID-19 cases in the context of low testing capacity, we may be underestimating the number of cases and their psychological burden on homemakers. Underdiagnosis in this population may be further compounded by their need to continue working for income, to leave the house to obtain food and other basics (delivery services were not available here), and to relieve overcrowding by spending time outside, thus deterring testing in cases where symptoms were mild. Despite this, the cumulative incidence of confirmed cases by the time of data collection for both waves in our study (5.8 per 100) is somewhat higher than that officially registered for the municipality where the villa is located (5226 per 100,000) [21].

### Study strengths and limitations

An important strength of this study is its longitudinal design and the wealth of data collected especially to assess covid-19 related stressors, allowing us to provide a multifaceted picture of the problem. Finally, its focus on peripheralized urban-poor homemakers, an underrepresented population, especially given the difficulties in reaching them in times of mobility restrictions is of especial value. However, there are limitations to our study. First, because of its longitudinal design, the telephone application of the survey during the pandemic implied an attrition of the study sample. As shown in Appendix Table 1, the distributions of gender and educational level have remained consistent since baseline in 2019. However, wave 4 introduced changes in the age distribution: participants aged 45–64 represented 61% of the sample in wave 4, compared with 46–47% in previous waves. This shift occurred at the expense of both younger and, especially, older participants. Importantly, women homemakers within the 45–64 age group are most likely to experience increased household burdens during lockdown, representing the primary focus of interest of our study. In fact, previous data have shown that in Chile, female-headed households were the most affected by the employment crisis during the COVID-19 pandemic. In addition, some analyses were conducted on smaller subsamples, as the corresponding determinants required specific household characteristics that were not present in all cases. This was particularly true when assessing the impact of students in the household experimenting difficulties with schoolwork completion, where analysis was restricted to households with school-aged children and youth (*n* = 207). Although this reduced the statistical power of the analysis, it did not compromise the ability to identify associations with homemakers’ mental health.

Second, despite the availability of previous measures of most of the independent and outcome variables in this study, we chose not to use them for two main reasons. One is the shift to telephone application of the survey, which limits comparability with previous waves. The other factor was the October 2019 social outburst—a three-month period of intense street protests, unprecedented since Chile’s return to democracy in 1989 [85]—which significantly affected our study population and undermined the suitability of the previous survey wave as a valid pre-COVID-19 baseline [86]. We thus introduced “perceived mood decline” as a dynamic measure to complement the assessment of symptoms of depression, offering some insight into whether the observed associations may reflect a causal relationship. The analysis of perceived mood decline largely supported the results for depressive symptoms. For example, the strong association between students failing to complete schoolwork in w4 is accompanied by a strong association with mood decline (1.47; 1.16–1.86), and the reduction in this association in w5 is accompanied by a lack of association with the latter (0.96; 0.53–1.89).

Third, most measures are self-reported, which may introduce measurement error—potentially underestimating associations. For example, as noted above, by restricting our measure to confirmed COVID-19 cases in a context of limited testing capacity, we may have underestimated the psychological burden of having a COVID-19 case within the household. Conversely, self-report may also introduce bias, potentially leading to an overestimation of associations: individuals experiencing depressive symptoms may perceive and report their circumstances as more problematic than those without such symptoms. However, we observe that not all associations between self-reported perception-based variables and depressive symptoms were significant.

Fourth, some survey items had to be modified between w4 and w5, limiting between-wave comparability (e.g., indebtedness). Finally, there were no reported COVID-19 deaths in our sample, so we could not observe the mental health consequences of bereavement, the normal course of which was altered by mobility restrictions, hampering the implementation of culturally adequate coping strategies, such as memorial services [13, 87]. Bereavement can represent a significant public health problem, the burden of which is estimated to be several times greater than the death toll [88]. Further research is necessary, especially among peripheralized populations with limited access to specialized health services, to implement the support systems these populations need to cope with disease and death in contexts of crisis.

### Implications

Lessons drawn from the COVID-19 crisis may support decision makers in future pandemics and in other extreme events, such as armed conflicts and major natural disasters. To do so, however, as noted by Arendt et al. [89], analysing and understanding the impact of nonpharmacological containment measures is highly important. As recommendations derived from earlier experiences, the Lancet Commission on lessons for the future from the COVID-19 pandemic highlights that “ensuring mental health and well-being is a crucial part of preparedness and response to pandemics and other similar threats” [90]. On the other hand, the OECD [90] highlights the need to redefine the social contract, with stronger social protection systems and putting well-being and sustainability at the centre, as one of the key lessons from COVID-19.

Our study underscores the need for remote schooling preparedness, including maternal school support, family-based services and support for educators, especially for peripheralized, low-income students, and support for children wellbeing [91. On the other hand, as the pandemic made visible, our results highlight the need to expand social security to include mechanisms to provide coverage to all, including informal workers, in the context of mobility restrictions to ensure families’ continuity of income.

Differences and similarities across studies in and beyond the region provide a general framing to our findings, but comparisons must be made with caution given the heterogeneity of designs, pre-pandemic conditions and pandemic responses. The impact of the pandemic on wellbeing and mental health was highly contingent on context, and hence the centrality of the social determinants of health approach. Future research should delve further into the multiple forms of economic constraint faced by families in the context of economic crises; the developmental consequences for children and youth; and the impacts of infection, hospitalization and bereavement on mental health, and into the long-lasting effects of COVID-19 on mental health.

## Conclusions

Several COVID-19-related social determinants of health affected peripheralized populations and were associated with their mental health, particularly among women, younger individuals, and those facing socioeconomic adversity. Our findings underscore the multifactorial nature of COVID-19-related stressors in Chile, including housing, economic, and family-level determinants associated with increased symptoms of depression.

In our study, financial stressors, including job and income loss, unemployment, problematic indebtedness and food insecurity, were associated with symptoms of depression both during and after lockdown. Notably, the strongest determinant of poor mental health among homemakers during lockdown was the presence of household students experiencing difficulties completing schoolwork, a novel and policy-relevant finding in the Chilean context.

The lessons learned from the COVID-19 crisis must not go unattended, but should inform future policy responses to complex crises. This study contributes to that effort by offering insights for public policy aimed at supporting peripheralized urban-poor populations, helping to prevent or mitigate the deepening of existing social inequalities, and guiding future research on mental health and its social determinants.

## Supplementary Information

Supplementary Material 1.

## Data Availability

The datasets analysed during the current study cannot be shared openly to protect the privacy of participants and due to informed consent restrictions but are available from the corresponding author upon reasonable request.

## Abbreviations

RUCAS: “Regeneración Urbana, Calidad de Vida y Salud” project
w4: wave 4 of measurement (August 11–September 17, 2020)
w5: wave 5 of measurement (January 07 to February 12, 2021)
PHQ-2: Patient Health Questionnaire–2
