# Supplementary material for: Learning the hard way: What COVID-19 teaches us about the social determinants of mental health among the urban poor
Source: BMC Public Health. 2026 Jan 9;26:492. doi: 10.1186/s12889-025-25815-1 (PMC12882193; doi:10.1186/s12889-025-25815-1)
Supplement: Supplementary file 1 — Supplementary Material 1. [file 12889_2025_25815_MOESM1_ESM.docx]

## APPENDIX

Appendix Table 1. Sociodemographic characteristics of the baseline RUCAS sample, wave 3 sample (sampling frame for w4), and the study sample characteristics of homemakers.

|  |  | Baseline RUCAS sample | | Study sampling frame  (W3 sample) | | Study sample  (W4-W5) | |
| --- | --- | --- | --- | --- | --- | --- | --- |
|  |  | N=718 | | N=641 | | N=413 | |
|  |  | n | % | n | % | n | % |
| Sex | Men | 109 | 15.2 | 95 | 14.8 | 61 | 14.8 |
|  | Women | 609 | 84.8 | 546 | 85.2 | 352 | 85.2 |
| Age group (years) | 18-24 | 37 | 5.2 | 27 | 4.2 | 14 | 3.4 |
|  | 25-44 | 217 | 30.2 | 185 | 28.9 | 101 | 24.5 |
|  | 45-64 | 330 | 46.0 | 303 | 47.3 | 251 | 60.8 |
|  | ≥ 65 | 134 | 18.7 | 126 | 19.7 | 47 | 11.4 |
| Age (years) | Mean (SD) | 48.1 (13.7) | | 48.9 (13.4) | | 49.9 (12.3) | |
| Completed years of formal education | < 4 | 56 | 7.8 | 54 | 8.4 | 32 | 7.7 |
|  | 4 to 7 | 139 | 19.4 | 125 | 19.5 | 84 | 20.3 |
|  | 8 to 12 | 471 | 65.6 | 420 | 65.5 | 270 | 65.4 |
|  | > 12 | 41 | 5.7 | 31 | 4.8 | 19 | 4.6 |
|  | Missing | 11 | 1.5 | 11 | 1.7 | 8 | 1.9 |

Appendix Table 2. Prevalence ratio (95% CI) adjusted by covariates.

|  |  | **W4** | | | | | | |  | **W5** | | | | | | |
| --- | --- | --- | --- | --- | --- | --- | --- | --- | --- | --- | --- | --- | --- | --- | --- | --- |
|  |  |  | **Symptoms of depression** |  |  |  | **Mood decline** |  |  |  | **Symptoms of depression** |  |  |  | **Mood decline** |  |
|  |  | **Prevalence** | **PR** | **95% CI** |  | **Prevalence** | **PR** | **95% CI** |  | **Prevalence** | **PR** | **95% CI** |  | **Prevalence** | **PR** | **CI 95%** |
| **Household load of domestic and unpaid care work** | | |  |  |  |  |  |  |  |  |  |  |  |  |  |  |
| Toddlers (≤ 2 years) | No | 28.0% |  |  |  | 59.4% |  |  |  | 28.7% |  |  |  | 27.1% |  |  |
|  | Yes | 39.6% | 1.27 | 0.86 - 1.89 |  | 60.4% | 0.96 | 0.75 - 1.24 |  | 27.3% | 0.94 | 0.51 - 1.71 |  | 18.8% | 0.75 | 0.35 - 1.59 |
| Childen (< 15 years) | No | 27.0% |  |  |  | 62.9% |  |  |  | 28.0% |  |  |  | 26.5% |  |  |
|  | Yes | 32.3% | 1.08 | 0.75 - 1.56 |  | 56.7% | 1.05 | 0.88-1.26 |  | 29.3% | 1.02 | 0.71 - 1.49 |  | 22.8% | 0.89 | 0.61 - 1.29 |
| Older adults (> 64 years) * | No | 29.2% |  |  |  | 58.7% |  |  |  | 27.3% |  |  |  | 22.8% |  |  |
|  | Yes | 30.0% | 1.29 | 0.83 - 2 |  | 63.8% | 1.21 | 0.98-1.5 |  | 33.7% | 1.36 | 0.89 - 2.07 |  | 32.5% | 1.09 | 0.7 - 1.67 |
| Students´ school-work completion** | Achieved | 17.2% |  |  |  | 49.5% |  |  |  | 26.7% |  |  |  | 25.2% |  |  |
|  | Not achieved | 42.9% | 2.63 | 1.59 - 4.35 |  | 72.3% | 1.44 | 1.14 - 1.83 |  | 40.0% | 1.57 | 0.98 - 2.52 |  | 24.4% | 0.91 | 0.49 - 1.7 |
| **Relationships between household members** | | |  |  |  |  |  |  |  |  |  |  |  |  |  |  |
| Live alone | No | 30.8% |  |  |  | 60.6% |  |  |  | 29.3% | 1.00 |  |  | 25.8% | 1.00 |  |
|  | Yes | 18.8% | 0.68 | 0.27 - 2.46 |  | 51.1% | 0.92 | 0.67 - 1.27 |  | 22.9% | 0.84 | 0.47 - 1.51 |  | 31.3% | 1.02 | 0.61 - 1.7 |
| Overcrowding | No | 28.2% |  |  |  | 57.9% |  |  |  | 27.2% |  |  |  | 26.1% |  |  |
|  | Yes | 35.0% | 1.18 | 0.8 - 1.73 |  | 68.3% | 1.13 | 0.93 - 1.38 |  | 37.5% | 1.43 | 0.97 - 2.09 |  | 29.1% | 1.15 | 0.74 - 1.8 |
| Being able to hold a private conversation*** | Always/almost always | 26.1% |  |  |  | 55.0% |  |  |  | 19.3% |  |  |  | 20.7% |  |  |
|  | Sometimes | 27.6% | 1.02 | 0.62 - 1.65 |  | 70.7% | 1.22 | 0.97 - 1.52 |  | 18.2% | 0.94 | 0.42 - 2.07 |  | 27.3% | 1.30 | 0.68 - 2.49 |
|  | Never/almost never | 32.8% | 1.23 | 0.88 - 1.73 |  | 60.8% | 1.10 | 0.92 - 1.32 |  | 35.3% | 1.76 | 1.19 - 2.61 |  | 28.8% | 1.43 | 0.97 - 2.12 |
| Conflicts over space*** | Never/almost never | 26.1% |  |  |  | 57.7% |  |  |  | 24.3% |  |  |  | 27.9% |  |  |
|  | Sometimes | 35.6% | 1.31 | 0.83 - 2.05 |  | 53.3% | 0.89 | 0.67 - 1.18 |  | 29.3% | 1.26 | 0.8 - 2 |  | 20.7% | 0.78 | 0.45 - 1.35 |
|  | Always/almost always | 39.5% | 1.38 | 0.97 - 1.98 |  | 72.8% | 1.20 | 1.01 - 1.42 |  | 43.4% | 1.76 | 1.23 - 2.5 |  | 23.7% | 0.89 | 0.56 - 1.41 |
| **Employment during COVID-19** | | |  |  |  |  |  |  |  |  |  |  |  |  |  |  |
| Employment trajectory | Always occupied | 28.0% |  |  |  | 56.6% |  |  |  | 26.7% |  |  |  | 21.9% |  |  |
|  | Found a job | 22.2% | 1.02 | 0.29 - 3.6 |  | 33.3% | 0.68 | 0.27 - 1.73 |  | 22.4% | 0.89 | 0.51 - 1.54 |  | 19.3% | 0.87 | 0.47 - 1.63 |
|  | Loss a job | 39.5% | 1.38 | 0.95 - 2.01 |  | 69.4% | 1.21 | 1 - 1.48 |  | 34.8% | 1.35 | 0.85 - 2.16 |  | 37.0% | **1.63** | **1.01 - 2.64** |
|  | Never occupied | 28.1% | 1.00 | 0.7 - 1.45 |  | 59.9% | 1.04 | 0.86 - 1.27 |  | 29.5% | 1.06 | 0.73 - 1.56 |  | 30.8% | 1.31 | 0.87 - 1.97 |
| Employment status | Occupied who didn't lose income | 1.0% |  |  |  | 51.6% |  |  |  | 18.4% |  |  |  | 21.6% |  |  |
|  | Occupied who lost income | 33.8% | 1.66 | 0.97 - 2.85 |  | 61.0% | 1.25 | 0.93 - 1.69 |  | 36.3% | 1.97 | 1.23 - 3.14 |  | 20.3% | 0.96 | 0.56 - 1.67 |
|  | Unemployed | 40.0% | 1.90 | 0.96 - 3.76 |  | 55.0% | 1.11 | 0.73 - 1.7 |  | 35.0% | 1.86 | 0.92 - 3.72 |  | 25.0% | 1.27 | 0.55 - 2.94 |
|  | Out of the labour force | 30.4% | 1.42 | 0.87 - 2.34 |  | 62.7% | 1.24 | 0.96 - 1.61 |  | 30.9% | **1.60** | **1.03 - 2.5** |  | 32.6% | 1.43 | 0.94 - 2.18 |
| **Household financial situation** | | |  |  |  |  |  |  |  |  |  |  |  |  |  |  |
| Indebtedness**** | No debts | 26.2% |  |  |  | 55.1% |  |  |  |  |  |  |  |  |  |  |
|  | No new debts | 27.4% | 1.06 | 0.72 - 1.54 |  | 65.0% | 1.17 | 0.97 - 1.4 |  |  |  |  |  |  |  |  |
|  | Acquired new debts | 39.5% | 1.48 | 1.04 - 2.11 |  | 64.0% | 1.16 | 0.95 - 1.42 |  |  |  |  |  |  |  |  |
| Problematic indebtedness***** | No debts |  |  |  |  |  |  |  |  | 25.9% |  |  |  | 25.9% |  |  |
|  | High payment capacity |  |  |  |  |  |  |  |  | 27.8% | 0.99 | 0.59 - 1.64 |  | 34.0% | 1.21 | 0.78 - 1.87 |
|  | Low payment capacity |  |  |  |  |  |  |  |  | 38.6% | 1.44 | 1.01 - 2.07 |  | 24.3% | 0.99 | 0.62 - 1.56 |
| Financial distress***** | Little/none |  |  |  |  |  |  |  |  | 21.0% |  |  |  | 23.1% |  |  |
|  | Moderate |  |  |  |  |  |  |  |  | 24.2% | 1.35 | 0.85 - 2.16 |  | 24.2% | 1.63 | 1.01 - 2.64 |
|  | High/severe |  |  |  |  |  |  |  |  | 49.4% | 1.06 | 0.73 - 1.56 |  | 36.5% | 1.31 | 0.87 - 1.97 |
| Food insecurity | No | 26.4% |  |  |  | 58.3% |  |  |  | 26.5% |  |  |  | 25.6% |  |  |
|  | Yes | 49.1% | 1.88 | 1.35 - 2.62 |  | 69.8% | 1.21 | 0.98 - 1.48 |  | 41.4% | 1.61 | 1.13 - 2.28 |  | 31.6% | 1.22 | 0.79 - 1.9 |
| **COVID-19 infection** | | |  |  |  |  |  |  |  |  |  |  |  |  |  |  |
| Homemaker | No case | 29.7% |  |  |  | 59.8% |  |  |  | 29.1% |  |  |  | 26.4% |  |  |
|  | Case | 25.9% | 0.84 | 0.43 - 1.64 |  | 53.8% | 0.86 | 0.61 - 1.23 |  | 9.1% | 0.30 | 0.05 - 1.93 |  | 27.3% | 0.99 | 0.38 - 2.6 |
| Household member | No cases | 29.5% |  |  |  | 59.5% |  |  |  | 29.2% |  |  |  | 26.2% |  |  |
|  | Cases | 28.3% | 0.91 | 0.56 - 1.49 |  | 60.0% | 0.98 | 0.76 - 1.25 |  | 17.4% | 0.61 | 0.25 - 1.49 |  | 30.4% | 1.18 | 0.62 - 2.25 |

* Only homemakers aged less than 65 years and not living alone are considered (n= 357).

** Only households with school students are considered (n=207- missing data).

*** Only households with more than one member (n=365 – missing data).

**** Variable available only in w4.

***** Variables available only in w5.
